# A network analysis of difficulties in emotion regulation, anxiety, and depression for adolescents in clinical settings

**DOI:** 10.1186/s13034-023-00574-2

**Published:** 2023-02-22

**Authors:** Qian-Nan Ruan, Yu-Hsin Chen, Wen-Jing Yan

**Affiliations:** 1 Wenzhou Seventh People’s Hospital, Wenzhou, 325006 China; 2 School of Mental Health, Wenzhou Medical University, Wenzhou, 325035 China; 3 Zhejiang Provincial Clinical Research Center for Mental Disorders, The affiliated Wenzhou Kangning Hospital, Wenzhou Medical University, Wenzhou, 325035, China

**Keywords:** Emotion regulation, Network analysis, Anxiety, Depression, Adolescent

## Abstract

**Background:**

Difficulties in emotion regulation (DER) are widely considered to underlie anxiety and depression. Given the prevalence of anxiety and depression in adolescents and the fact that adolescence is a key period for the development of emotion regulation ability, it is important to examine how DER is related to anxiety and depression in adolescents in clinical settings.

**Methods:**

In the present study, we assessed 209 adolescents in clinical settings using the Difficulties in Emotion Regulation Scale (DERS) and the Hospital Anxiety and Depression Scale (HADS) and examined the associations between six components of DER and 14 symptoms of anxiety and depression. We used network analysis, constructed circular and multidimensional scaling (MDS) networks, and calculated network centrality, bridge centrality, and stability of centrality indices.

**Results:**

The results showed that: (1) The global centrality index shows that the Strategy component (i.e., lack of access to strategies) is the center in the whole network, ranking highest in strength, closeness, betweenness, and expected influence. (2) The MDS network showed a closeness of anxiety and depression symptoms, while Awareness component (i.e., lack of emotional awareness) stayed away from other DER components, but Awareness is close to some depression symptoms. (3) The bridge nodes of three groups, Strategy from DERS, Worry and Relax from anxiety symptoms, and Cheerful and Slow from depression symptoms, had the strongest relationships with the other groups.

**Conclusion:**

Lack of access to strategies remains in the center not only in DER but also in the DER-anxiety-depression network, while lack of awareness is close to depression but not to anxiety. Worrying thoughts and inability to relax are the bridging symptoms for anxiety, while lack of cheerful emotions and slowing down are the bridging symptoms for depression. These findings suggest that making emotion regulation strategies more accessible to patients and reducing these bridging symptoms may yield the greatest rewards for anxiety and depression therapy.

## Background

### Two popular frameworks for emotion regulation

Emotion regulation (ER) is a multidimensional construct that has been proposed to encompass different ER strategies and difficulties with ER [3]. The two perspectives give rise to two distinct theoretical frameworks with corresponding measures.

One framework based on emotion science views emotion regulation as a heterogeneous set of actions designed to influence “which emotions we have, when we have them, and how we experience and express them” [19]. The nature of ER is still left with much confusion, given the elusive definition of “emotion” [20]. What emotions one has and how they are expressed are influenced by the type and timing of the emotion regulation strategy an individual uses [53]. Gross’s process model proposed five points or stages in the emotion generative process at which emotions can be regulated, include situation selection, situation modification, attentional deployment, cognitive change, and response modulation [42]. The Emotion Regulation Questionnaire (ERQ) was developed on this framework with the aim to assess individual differences with primarily concerning discrete strategies for modulating emotional experience: reappraisal and suppression [18].

Another framework of ER, concerning trait-level abilities, proposed four broad facets of emotion regulation by Gratz and Roemer [15],: (a) awareness and understanding, (b) acceptance, (c) the ability to control impulses and follow target behaviors in the presence of negative emotions, and (d) emotion regulation strategies are effective in enhancing the emotional environment. The DERS is considered a comprehensive measure of difficulties in ER and has often been used in research within clinical and treatment settings [21]. Based on this theory, the Difficulties in Emotion Regulation Scale (DERS) was then developed to assess emotion regulation [15], including six subscales: (1) lack of emotional awareness; (2) lack of emotional clarity; (3) difficulty regulating behavior when distressed; (4) difficulty engaging in goal-directed cognition and behavior when distressed; (5) unwillingness to accept certain emotional responses; and (6) lack of access to strategies for feeling better when distressed [21].

DERS has been used more in clinical contexts whilst ERQ has been more commonly used in college samples [52]. These differing descriptions of the measures by the scale developers are notable, as Gratz and Roemer [15] developed the DERS, in large part, to extend the assessment of emotion dysregulation beyond assessing for specific emotion regulation strategies. More precisely, and as noted above, Gratz and Roemer [15] asserted that subjective appraisal of one’s ability to effectively regulate emotions is particularly important when considering the role emotion dysregulation in psychopathology. It seems clear that the construct of emotion dysregulation, in its entirety, must account not only for specific strategies, but also for other processes which impact emotional responding (e.g., identification and understanding of emotions). With such considerations, the use of a more context-dependent measure of emotion regulation difficulties, such as the DERS, may offer advantages over more limited examinations of specific emotion regulation strategies (e.g. cognitive reappraisal and expressive suppression from ERQ) in this study. In addition, DERS show promising internal consistency and validity in a community sample of different demographical groups [38, 45]. Many studies also show that results were consistent across both adolescent and adult samples [28, 59]. These findings provide evidence for the validity in clinical contexts and supply further evidence that emotion regulation difficulties are an excellent transdiagnostic marker of psychopathology risk.

### DER and emotion disorders in adolescents

Given the widespread anxiety and depression among adolescents, it is important to understand what contributes to symptoms of anxiety and depression.

A key period for the development of emotion control methods may occur during adolescence [24] and emotion regulation may progress concurrently with physical growth [51]. According to McRae et al. [31], brain development during adolescence may contribute to the development of emotion regulation techniques, indicating a complex interaction between biological and environmental factors. If a person has persistent DER, it can lead to psychosocial impairment [39]. DER represents a potential risk factor for anxiety and depressive symptoms and has been the subject of extensive empirical investigation.

Numerous studies have shown a significant positive correlation between DERS scores (especially total scores) and symptoms of various psychological disorders, including borderline personality disorder [16], Generalized Anxiety Disorder (GAD) [34], substance use disorder [17], social anxiety [47], health-related anxiety [3], posttraumatic stress disorder [9], and bipolar disorder [4, 58].

#### DER and anxiety

The link between anxiety and emotion dysregulation is common in the literature [8, 48]. Although many studies have found that adults with elevated anxiety symptoms engage in more nonacceptance, suppression, avoidance, worry, and rumination, fewer studies have examined such relationships among anxious adolescents [48]. Research found that dysregulation in emotion could influence the occurrence of anxiety disorders [8].One study found that anxious children displayed more difficulty in employing appropriate emotion regulation strategies than non-anxious children [7]. Weems [60] argued that anxiety disorders in children were best considered as difficulties in regulating anxiety syndromes and negative emotions in general (e.g., experiencing restlessness, worry). Previous research shows that children with anxiety disorders (and other internalizing disorders) tend to have poor emotion understanding, low self-efficacy about their ER abilities, and difficulty expressing certain emotions [48].

Many studies have examined the relationship between various types of anxiety and DERS, although the majority of subjects have been adults. Theoretical, experimental and clinical evidence suggests that patients with GAD are characterized by dysregulated emotion regulation strategies [2]. According to the emotion dysregulation model, GAD is characterized by a rapid, easy and high-intensity experience of emotions [33]. This kind of emotional response leads to difficulties in emotion regulation and is compounded by the difficulty of experiencing clear emotions in people with GAD. Another study showed that DER reliably predicted GAD and the experience of nonclinical panic attacks [55]. Another study also found that the severity of PTSD symptoms was linked to a lack of emotional acceptance, difficulty focusing on target behaviors when upset, difficulty controlling impulses, limited access to effective emotion regulation strategies, and a lack of emotional clarity [54].

#### DER and depression

Self-reported DER has been linked to depressive symptoms across adolescents [38]. Those with worse emotions and less effective emotion regulation reported more depressive symptoms and problematic behaviors [50]. Negative emotional expression, regarding difficulties in emotion regulation, has also been linked to depressive symptoms [41]. In addition, some studies have shown that DER longitudinally predicts depressive symptoms before adolescence [29]; in another longitudinal study found this to be true for over one year during early adolescence [22].

The relationships among DER components and depression in adolescents may be analyzed as follows [14]. Adolescents may not be aware of their emotions. As a result, they may not use emotion regulation because it inherently needs to know what to be modified. This can make adolescents experience negative emotions with persistence. Even when adolescents are aware and clear about their emotions, they may be unable to accept them. Then the adolescents can only repress the emotions, leading to their more intensive emotions.

Furthermore, negative emotions may make adolescents unable to be fully involved in goal-directed behaviors. Research has shown that those unable to have goal-directed behaviors in negative emotions could lead to depressive symptoms in adolescents [25]. Similarly, adolescents may have a rash, impulsive behaviors in response to negative emotions. Also, studies have shown that impulsivity can predict depressive symptoms in adolescents [62]. In addition, it has been suggested that adolescents with depressive symptom may not possess the strategies required to regulate negative emotions. Studies have shown that adolescents with MDD find it difficult to develop adaptive strategies and utilize them for regulatory purposes [1].

Altogether, these results make clear that various difficulties related to emotion regulation are associated with anxiety and depressive symptoms. Adolescents may experience persistent negative emotions with these problems, making them more likely to suffer from anxiety and depression [49]. The DER components and emotional symptoms can form a complex network, the analysis of which may facilitate our understanding of DER and anxiety & depression.

### Network analysis

Thus, network analysis was employed to analyze the relationships among DER components and symptoms of anxiety & depression. We worked bottom-up and did not apply any top-down constructs consistent with standard reductionist biomedical models. In an estimated network structure, the centrality indices represent the overall connectivity of a particular symptom (or component). The central node contributes most to the interconnectedness of the symptoms (or components) in the estimated network structure [6, 46]. When a highly central component is activated, it influences and activates other components, and thus considered to establish the network [57]. In other words, a tightly connected network with many strong connections among the symptoms is considered risky because activation of one symptom can quickly spread to others, leading to more chronic symptoms over time. In addition, we calculated the bridge centrality. Previous research has found that deactivating bridge nodes prevents the spread of comorbidity (i.e., one disorder activating another) [27].

In the present study, we characterized the network structure of DER components and symptoms of anxiety and depression for adolescents in clinical settings. We investigated the network structure and centrality indices, and then checked the stability of the centrality indices for the network, with the aim of gaining insights into the relationship between DERS and anxiety and depression symptoms, which will further expand our knowledge of ER mechanism in psychopathology (anxiety and depression), and provide clinical implications for anxiety and depression therapy.

## Method

### Participants

The sample consisted of 209 adolescents aged 12 to 18 years that sought clinical aid at a psychiatric hospital between February and May of 2022. Each individual completed the hospital registration procedure, followed by a one-on-one mental health assessment by the hospital psychiatrist. Prior to the mental-health assessment, patient and parents/guardians were briefed on the assessment procedure and scales used, followed by the signed informed consent from both parent/guardian and patient as required by local hospital ethical regulations. The patient was then led to an assessment room under the company of the hospital psychiatrist and asked to complete the Hospital Anxiety and Depression Scale (HADS) and the DERS in addition to the routine diagnosis process. When the patient was completed, the psychiatrist checked to ensure that all sections of the HADS and DERS had been completed. All procedures contributing to this work conformed to the ethical standards of the relevant national and institutional human experimentation committees, as well as to the 1975 Declaration of Helsinki (revised 2008). All procedures involved were approved by the IRB of the Seventh People's Hospital of Wenzhou (EC-KY-2022048).

### Measures

#### Hospital anxiety and depression scale (HADS)

The HADS assesses both anxiety and depression, which commonly coexist. The measure is employed frequently due to its simplicity, speed, and ease of use. Very few literate people have difficulty completing it. The HADS contains 14 items, including seven for depressive symptoms (i.e., the HADS-D) and seven for anxiety symptoms (i.e., the HADS-A), focusing on non-physical symptoms. The correlations between the two subscales vary from 0.40 to 0.74 (with a mean of 0.56). The Cronbach's alpha for the HADS-A varies from 0.68 to 0.93 (with a mean of 0.83) and for the HADS-D from 0.67 to 0.90 (with a mean of 0.82). The sensitivity and specificity for both is approximately 0.80 [5]. Many studies conducted around the world have confirmed that the measure is valid when used in a community setting or primary care medical practice.

#### Difficulties in emotion regulation scale (Chinese version)

The scale of DERS is a 36-item self-report measure. It contains six factors: nonacceptance of emotional responses, difficulty engaging in goal-directed behavior, impulse control problems, lack of emotional awareness, limited access to emotion regulation strategies, and lack of emotional clarity. Higher scores indicate more difficulty in emotion regulation. The Cronbach’s alpha of the scale and six subscales ranges from 0.88 to 0.96, and test–retest reliability from 0.52 to 0.77. Confirmatory factor analysis has shown the following fit indices: *X*^*2*^*/df* = 1.05, *AGFI* = 0.87, *GFI* = 0.90, *NFI* = 0.94, *CFI* = 0.99, *TLI* = 0.99, *NNFI* = 0.9, *RMSEA* = 0.015, and *KMK* = 0.06 [30].

### Network analysis

We constructed the network using Gaussian graphical models (GGMs) via the R package (R Core Team version 4.1.3) *qgraph* (version 1.9.2) [10]. GGMs estimate many parameters (i.e., 20 nodes need to estimate 190 parameters: 20 threshold parameters and 20 * 19/2 = 190 paired correlation parameters), likely leading to false positive edges. Therefore, GGMs are usually regularized by a graphical lasso, resulting in a sparse (i.e., resolved) network with as few edges as possible to explain the correlation or covariance between nodes [13]. The R package *qgraph* was used to calculate and visualize the networks. We also measured the centrality and stability of the established network. The R package *qgraph* and *estimateNetwork* automatically implement the glasso regularization, combined with an extended Bayesian information criterion (EBIC) model [12].

In network terminology, symptoms of anxiety, depression and DER components are “nodes” and the relationships among the nodes are “edges.” The edge between two nodes represents the regularized partial correlation coefficient, and the thickness indicates the association's magnitude. The graphical lasso algorithm makes all edges with small partial correlations shrink to zero. It thus facilitates the interpretation and establishment of a stable network, solving traditional lost-power issues that emerge from examining all partial correlations for statistical significance [11]. For the present network, we divided the components into three groups or communities: anxiety (seven symptoms), depression (seven symptoms), and DER (six components).

**Fig. 1:**
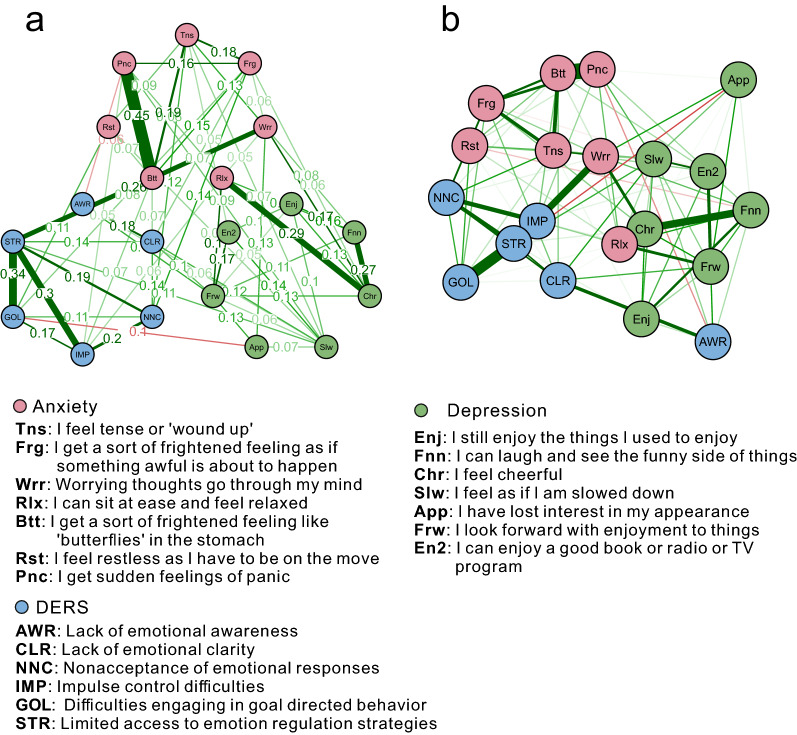
The network structure based on 209 adolescents. The network structure is a network of EBICglasso based on GGM. Green edges represent positive correlations, and red edges indicate negative correlations. The thickness of the edge reflects the magnitude of the correlation. **a** The network is with “circle” layout for easy viewing. It is important to note that the node positions don’t indicate Euclidean distances. The value (weight) on the edge indicates regression coefficient after regularization. **b** The network is with MDS, showing proximities among variables as distances between points in a low-dimensional space

Most network studies in psychopathology have used the FR algorithm to plot graphs, where the node locations don’t have meanings in space but to position them in a way that facilitates viewing network edges and clustering structures [27]. We used the "circle" layout for easier viewing, which places all nodes in a single circle, with each group (or community) put in separate circles (see Fig. 1a). In addition, we employed a multi-dimensional scaling (MDS) approach to display the network (see Fig. 1b). MDS expresses the proximity between variables as distances between nodes in a low-dimensional space, which is particularly useful for understanding networks, as the distances drawn between nodes can be interpreted as Euclidean distances [27].

*Centrality* We calculated several indices of node centrality to identify the most central symptom or component of the network. For each node, we calculated strength (i.e., the absolute value of the edge weights connected to the node), proximity (i.e., the average distance of the node from all other nodes in the network), spacing (i.e., the number of times a node lies on the shortest path between two other nodes), and expected influence (i.e., the sum of the edge weights connected to the node). In addition, node bridge strength is defined as the sum of the values of all edges connecting a particular node in a community to nodes in other communities; it is calculated with R-package *networktools* [27]. A higher node bridge strength value indicates more likely influence other groups.

*Stability of centrality indices* We investigated the stability of the centrality index by estimating a network model based on a subset of the data and a case-removal bootstrap (*N* = 1000). We considered the centrality index unstable if the correlation value decreased substantially with participant removal. The robustness of the network was assessed by the R-package *bootnet* using bootstrap methods [11]. This stability is calculated using the CS coefficient. It calculates the maximum proportion of cases with 95% certainty to retain correlations with raw centrality above 0.7 (default).

## Results

All the adolescents were included in the analysis. The students’ average age was 15.51 years (*SD* = 1.36). Descriptive statistics for these adolescents can be found in Table 1.

**Table 1:** Descriptive statistics for the measurements^1^

		*N*	DERS		Anxiety		Depression	
			*M*	*SD*	*M*	*SD*	*M*	*SD*
Sex	Female	98	122.34	27.93	11.04	5.01	10.40	5.19
	Male	108	92.29	25.11	6.69	4.81	6.40	4.84
Family structure	Regular	142	99.20	29.07	7.42	4.95	6.90	5.07
	Irregular^2^	27	118.67	27.87	11.56	5.47	10.70	4.65
Location	Urban	60	106.50	29.03	8.40	5.10	8.62	5.11
	Rural	109	100.01	29.92	7.90	5.34	6.90	5.14
Economic status	Wealthy	47	99.91	29.03	7.23	4.90	6.57	5.09
	Normal/poor	121	103.07	30.04	8.32	5.31	7.79	5.15
Age	12	3	134.00	32.92	12.33	4.73	13.00	6.08
	13	19	125.95	27.33	12.95	5.21	12.26	5.16
	14	21	124.76	28.30	11.81	5.92	9.29	5.99
	15	35	110.00	29.59	9.51	4.68	8.60	4.94
	16	81	95.06	28.11	7.25	4.99	7.07	5.12
	17	42	103.21	28.37	7.29	4.94	7.38	4.81
	18	3	124.33	1.53	8.67	4.73	7.33	4.04

Note that there is some missing information, making that the *N* does not always equals 209

Irregular includes single parent, reconstituted family and orphan

In the entire network, approximately 51.57% of all 190 network edges were set to zero by the EBICglasso algorithms. Figure 1 displays the network of DER components and anxiety and depression symptoms. Figure 1a displays an easily viewable circular network with weights on each edge. For example, the strongest edge (weight = 0.45) among the anxiety symptoms was between *Btt*^1^ (Butterfly: “I get sort of a frightened feeling, like butterflies in the stomach”) and *Pnc* (Panic: “I get sudden feelings of panic”). Among depression symptoms, the strongest edge (weight = 0.27) was between *Chr* (Cheerful: “I feel cheerful”) and *Fnn* (Funny: “I can laugh and see the funny side of things”). For DER components, the strongest edge (weight = 0.34) was between *STR* (“Limited access to emotion regulation strategies”) and *GOL* (Goal: “Difficulties engaging in goal-directed behavior”). Particularly, there was a strong connection (weight = 0.33) between *Rlx* (Relax: “I can sit at ease and be relaxed”) from the anxiety group and *Chr* from depression. Here, the weight indicates regression coefficient after regularization.

Figure 1b displays the MDS network. Highly-related nodes appear close together, whereas weakly-related nodes are farther apart. The node connections were closer within (vs. between) the anxiety, depression and DER communities, demonstrating their relative independence from one other. Among the DERS components, *AWR* (Awareness) stays apart from other components including *CLR* (Clarity), *STR* (Strategy), *GOL* (Goal), *IMP* (Impulse), and *NNC* (Non-acceptance). In the anxiety-depression network, some of the nodes were intertwined, such as *Rlx* (Relax: “I can sit at ease and be relaxed”) and *Chr* (Cheer: “I feel cheerful”), which echoes previous studies of anxiety and depression comorbidity that employed network analysis [43, 61].

### Centrality indices

**Fig. 2:**
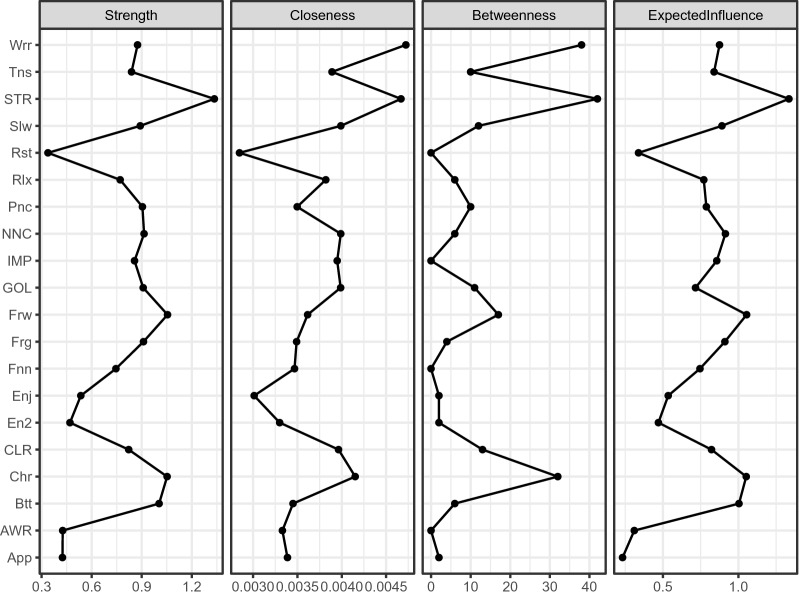
Centrality indices for the nodes of the present network, including those for strength, betweenness, closeness, and expected influence. The full names of the abbreviations can be found in Fig. 1

For the centrality indices, the values were scaled (i.e., normalized) relative to the highest value for each measure. Figure 2 shows the centrality indices, ordered by Expected Influence. *STR* (“Limited access to emotion regulation strategies”) from the DER components was the most central symptom, according to four indices. *Frw* (Forwrod: “I look forward with enjoyment to things”) from the depression symptoms and *Btt* (Butterflies: I get a sort of frightened feeling like 'butterflies' in the stomach) from the anxiety symptoms ranked high in their own groups, indicating that these nodes had strong relationships to the other nodes. For closeness and betweenness, *Wrr* (Worry: “Worrying thoughts go through my mind”) from the anxiety symptoms and *Chr* (Cheerful: “I feel cheerful”) from depression symptoms ranked the highest, meaning that they were closest to all other nodes in the network and usually stay between two other nodes.

### Bridge centrality indices

**Fig. 3:**
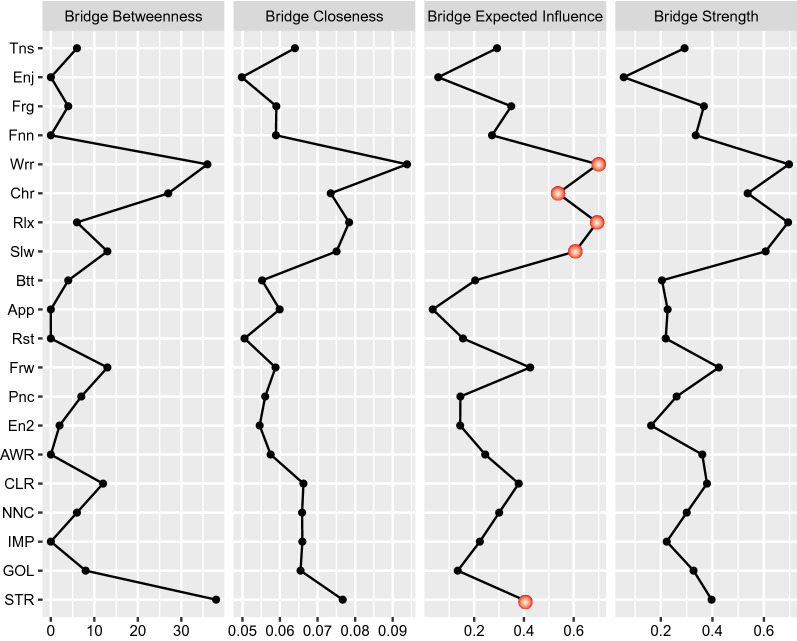
Estimated bridge centrality indices for the present network, including bridge strength, betweenness, closeness, and expected influence. The full names of the abbreviations for the nodes can be found in Fig. 1

We also calculated the bridge centrality indices (see Fig. 3). From four indices (i.e., bridge betweenness, closeness, expected influence, and strength) has the same meaning as mentioned in Session 3.1, but bridge centrality indices only show the influence on other groups. For example, *STR* (Strategy) in DERS ranked the highest, indicating these nodes had strong connections to anxiety and depression symptoms. *CLR* (Clarity) also has a relative strong connection to anxiety and depression symptoms. In other two groups, *Wrr* (Worry) and *Rlx* (Relax) from anxiety symptoms rank highest (strongest nodes connecting other groups) in among anxiety symptoms, and *Chr* (Cheerful) and *Slw* (Slow: I feel as if l am slowed down) from depression symptoms.

### Stability of the centrality indices

**Fig. 4:**
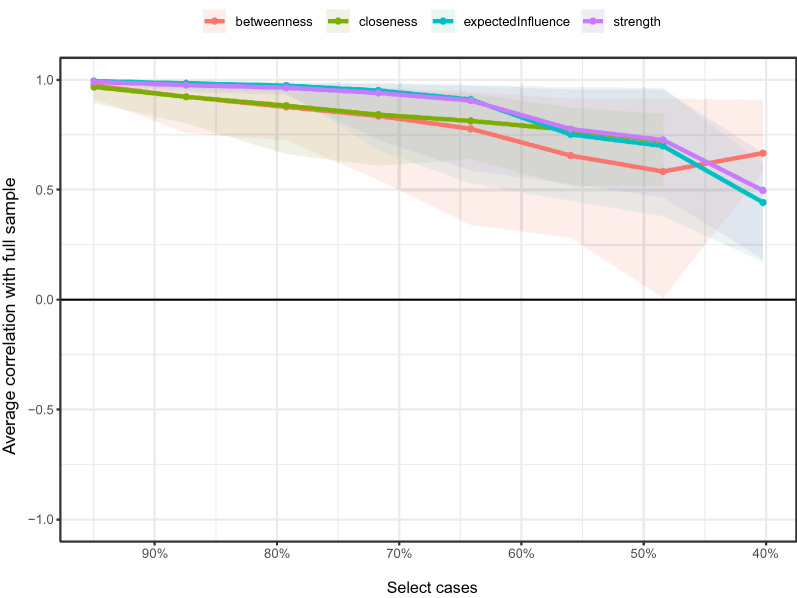
The average correlation coefficients between selected cases and the full sample for the centrality indices of networks. Lines indicate the means and areas ranging from the 2.5th quantile to the 97.5th quantile

Figure 4 shows the average correlations of centrality indices between the full sample and the selected cases. The rationale is to calculate maximum drop proportions to retain correlation of 0.7 in at least 95% of the samples. In this study, the CS coefficient indicated that the betweenness (CS(cor = 0.7) = 0.208), indicating that we can drop 20.8% of the samples to retain correlation of 0.7 with the full sample. For other indices, closeness (CS(cor = 0.7) = 0.208), strength (CS(cor = 0.7) = 0.283) and expected influence (CS(cor = 0.7) = 0.283). The CS coefficient value should preferably be above 0.5 and be at least 0.25. Therefore, strength and expected influence are relative more stable.

## Discussion

Depression and anxiety, widely viewed as the result of difficulties with emotion regulation [35], are becoming more severe in adolescents. Considering the relationship between DER and mood disorders, it is appropriate to put anxiety, depression, and DER components into a single network, where the DERS is the *ability* of ER while the anxiety and depression are the *symptoms*. An advantage of network analysis is that the discovery of dimensions does not require an a priori definition. Anchored in the network perspective, this study illustrated the node pathways, central indices, and central bridging indices for adolescents' DER and anxiety-depression networks. Therefore, we can infer the mechanism between the ER ability and Anxiety-depression symptoms.

Global centrality index shows Strategy (i.e., lack of access to strategies) is the center in whole network, ranking highest in strength, closeness, betweenness and expected influence. It should be noted that the Strategy subscale in DERS and strategies in ERS is different both in direction and content. According to Gratz and Roemer [15], Strategy is connected to “the belief that there is little that can be done to regulate emotions effectively once an individual is upset”, which makes it difficult to manage and control emotions [40]. Effective emotion regulation strategies may be especially important during adolescence, due to the increase in stress and rate of depression during this period. A meta-analysis has shown that that emotion regulation strategies are related to life satisfaction, positive affect, depression, anxiety, and negative affect [26, 32]. Research has shown that the ability to regulate one’s emotions is a key target intervention across various mental health presentations [36], and cognitive behavioral therapy for emotional disorders often relies on emotion regulation strategies to be successful [44]. As one study has found that participants diagnosed with at least one anxiety disorder or MDD who report high DERS scores still manage to effectively reduce induced negative emotions in 3 min when required to use emotion strategies [37]. Further, as the bridge centrality indices shows, the Strategy and Worry ranks the highest in connecting to the other sub-networks. Strategy is strongly related to Worry in anxiety sub-network, while Worry is strongly connected to the symptoms in anxiety and depression. Therefore, Strategy is the key node in DERS and making it more accessible to it may have positive influence to anxiety and depression (through Worry). It is reasonable for clinical practitioners not only to instruct patients with new emotion regulation strategies, but also encourage them to pay attention to and engage with optimal individual strategies when faced with Worry (“Worrying thoughts go through my mind”).

In addition, MDS approach (showing in Fig. 1b) allows for a more detailed representation of the interrelationships between items and the mutual positioning. These three sub-networks of anxiety, depression and DERS are relative independent with each other, but there are some intertwines, indicating the inner connections. The use of MDS approach should be encouraged in the future as it indicates the spatial relationships of nodes. According to the positioning in Fig. 1b, the Awareness is far away from other abilities in DERS, which is echoed by previous research [3, 15, 21] but stay close to the item of Enjoy (“I still enjoy the things l used to enjoy”) and Forword (“I look forward with enjoyment to things”) in Depression sub-network, which seems to indicate difficulties in awareness of one’s emotion may maintain the symptoms such as lack of enjoyment and thus maintain depression. Such results were inconsistent with previous research, which showed no direct relationship between emotional awareness and depressive symptoms, though emotional awareness yielded a significant mediation effect through total adaptive ER strategies on higher depressive symptoms [56]. In addition, the Awareness from DERS was not significantly associated with any of the symptoms of anxiety, supported by some (but not all) of previous research show “With the exception of ‘lack of emotional awareness’, social anxiety disorders participants reported significantly higher levels of ER difficulties when controlling for depression” [23].

### Limitations

Several limitations of this study will guide our future work. First, a cross-sectional design was used to construct DER and anxiety-depression networks. Therefore, the present study cannot be used to determine whether anxiety and depression symptoms cause DER components and vice versa. Future work will use a longitudinal approach with repeated measures of anxiety-depression and DER components to clarify the causal relationship between the two.

Second, the potential pathways found between the components may be limited to the DERS and HADS scales used. When we change the framework of emotion regulation from the clinical situational model to basic emotion science, the network structure is quite different. This diversity may lead to different findings and even some contradictory results.

Third, the sample size of this study was only 209 from the local psychiatric hospital. Future studies should increase the sample size and its variability to obtain a more comprehensive and stable network structure for more reliable conclusions.

Fourth, more scales than anxiety and depression should be included. Comorbidity is very common in psychopathological disorders, so symptoms such as subjective feelings of restlessness and impulsivity may also be found in other disorders such as ADHD. With more assessments, we will have a better understanding of the mechanisms of emotional dysfunction.

## Conclusions

The present study adds to the literature on how anxiety and depressive symptoms are related to DER components in adolescents in clinical settings. Lack of access to strategies stays in the center not only in DER but also in the network of DER-anxiety-depression. Lack of awareness is close to depression but not to anxiety. Worrying thoughts and inability to relax are the bridging symptoms for anxiety, while lack of cheerful emotions and slowing down are the bridging symptoms for depression. These findings suggest that reducing these bridging symptoms through therapy for anxiety and depression may yield the greatest benefits by making emotion regulation strategies more accessible to patients.

## Footnotes

1 Following, the node labels with abbreviations will be in italics.

## Declarations

### Data Availability

The raw data supporting the conclusion of this article is available, without undue reservation. https://osf.io/2mdqh/
